# End-Effector Technologies for Fruit Harvesting Robots: A Review of Structures, Actuation, and Field Deployability

**DOI:** 10.3390/s26113382

**Published:** 2026-05-26

**Authors:** Senming Zhong, Chen Shu, Liancai Shen, Zhangjun Wu, Minglong Xue, Xiaojun Wang, Weiwei Zhu

**Affiliations:** 1 Industrial Training Centre, Guangdong Polytechnic Normal University, Guangzhou 510665, China; itcsmzhong@gpnu.edu.cn (S.Z.); shuchen@stu.gpnu.edu.cn (C.S.); wuzhangjun@gpnu.edu.cn (Z.W.); weiweizhu@gpnu.edu.cn (W.Z.); 2 School of Mechanical and Electrical Engineering, University of Electronic Science and Technology of China, Chengdu 611731, China; 202521040136@std.uestc.edu.cn; 3 College of Computer Science and Engineering, Chongqing University of Technology, Chongqing 400054, China; xueml@cqut.edu.cn

**Keywords:** agricultural harvesting robot, end-effector, actuation, unstructured environment, field evaluation, review

## Abstract

This review summarizes the research on the end effectors of agricultural harvesting robots (2010–2025) and extracts two core design principles. First of all, the selection of end effectors must follow the biological characteristics of fruits: rigid grippers are suitable for hard skinned and regular fruits; soft grippers can reduce the damage of fragile crops to a certain extent; suction cups are suitable for smooth, barrier free surfaces; the envelope type is suitable for soft and lossless picking scenes; the combined suction and grip design is more suitable for unstructured environments. Secondly, the separation mode should match the characteristics of the stem: motion separation (torsion/pull) is suitable for weak stems, while cutting is mainly used for hard stems. Unlike previous literature, this review provides a field deployability checklist (including dust/water proofing, cleanliness, maintenance, aging prevention, and aspiration prevention) to narrow the results of the laboratory and the real field environment. The three future directions of multimodal perception, variable stiffness driving and reinforcement learning are logically related to the analysis in this paper: multimodal perception optimizes the perception limit, variable stiffness solves the rigid–flexible trade-off, and reinforcement learning provides adaptive strategies for different crops. This framework can match the end effector design with the crop-specific field conditions.

## 1. Introduction

With the continued growth of the global population and the increasing demand for food, coupled with the practical challenges of labor shortages, an aging population, and rising labor costs, the transformation of traditional agriculture toward automation has become an inevitable trend [1]. Fruit and vegetable harvesting, as one of the most labor-intensive operations, accounts for a significant portion of total harvesting time and is characterized by pronounced seasonality, high time sensitivity, strong repetitiveness, and substantial labor intensity [2,3,4]. Research has shown that traditional manual harvesting is not only inefficient but also prone to fruit damage caused by fatigue during operation, leading to economic losses [5]. Therefore, developing end-effectors capable of adapting to a wide variety of fruits and unstructured environments is a core direction for resolving the above-mentioned supply–demand contradiction and achieving agricultural modernization [6,7].

In an agricultural harvesting robot system, the end-effector is the mechanism mounted at the end of the robotic arm that is responsible for direct physical interaction with the crop or fruit, and it directly affects system performance [8]. Unlike industrial robots, which have fixed grasping positions and rigid workpieces, agricultural objects exhibit biological heterogeneity in terms of crop shape, size, and maturity, as well as spatial position uncertainty, and the fruit peel is generally fragile and easily damaged [9,10]. These biological characteristics make the design of the end-effector a key technological bottleneck limiting the commercial application of harvesting robots [11]. The key is how to design a system end-effector mechanism that can achieve a high efficiency and success rate while maintaining a low fruit damage rate [12,13].

Recent years have seen the end-effector evolve well beyond a simple mechanical gripping mechanism, integrating multimodal sensing, intelligent decision-making, and flexible actuation into soft robotics, machine vision, and artificial intelligence [14,15,16]. The findings indicate that existing research has developed several distinct technical routes. Traditional rigid grippers, despite their well-established control methods, appear to struggle with regulating gripping force, remaining prone to fruit damage [17,18]. The envelope mechanism that imitates human hand grasping reduces the risk of fruit damage by increasing the contact area and decreasing the distributed pressure, but it is difficult to apply to fruits of different sizes [19,20]. Furthermore, suction-based devices utilizing vacuum principles offer clear advantages in damage-free operation, but important requirements are put forward for the surface characteristics of fruits and the stability of picking posture [21,22]. In addition, for special crop morphologies or complex operational scenarios, researchers have further proposed various optimization schemes such as dual-arm collaborative operation [23,24], deep learning-based visual guidance [25,26,27], and bionic flexible structures [28,29]. The above technical routes each focus on different trade-offs among the three core indicators of efficiency, success rate, and damage rate, and their designs are often customized and optimized for specific crops [30,31]. Therefore, faced with a wide variety of crops, existing end-effectors still exhibit a significant trade-off challenge between cross-species versatility and single-crop operational efficiency.

Existing articles have systematically sorted out the design of end effectors and crop-specific applications but rarely touched on the fundamental problem that it is difficult to effectively compare data. For example, the success rate, damage rate and picking time data are usually reported under the condition of a large gap (for example, laboratory bench and commercial orchard, sheltered and non-sheltered, single fruit and fruit string, etc.), which is difficult to effectively compare. This paper first summarizes the existing research results, and then critically examines the effectiveness of comparison in different environments, and proposes a field deployability checklist to narrow the gap and initially achieve standardization.

The present review adds three principal contributions beyond earlier work. First, it undertakes a systematic quantitative comparison encompassing more than ten crop varieties, assessing grasping success rates, fruit damage percentages, and cycle times in parallel. Second, in light of constraints encountered in actual field operations—namely protection against dust ingress, cleaning and sanitization protocols, and material aging or deterioration—it formulates a field deployability checklist. Third, it constructs a decision look-up table that directly links fruit morphological traits with the most suitable end-effector configurations. The systematic retrieval strategy is detailed in Appendix A.

## 2. Operational Mechanisms, Interaction Modes, and Driving Methods of End-Effectors

The agricultural harvesting process is a complex engineering system involving perception and recognition of unstructured environments, robotic arm path planning, and end-effector operation. Among them, the operational process of the end-effector is typically decomposed into four core stages: target approach, stable grasping, fruit detachment, and fruit release. Grasping and detachment are the key links that directly determine the success or failure of harvesting. Therefore, this chapter will review existing technologies from two dimensions: grasping mechanism and detachment mode, based on the interaction mode between the mechanical system and the biological organism.

### 2.1. Grasping Mechanisms

**Figure 1:**
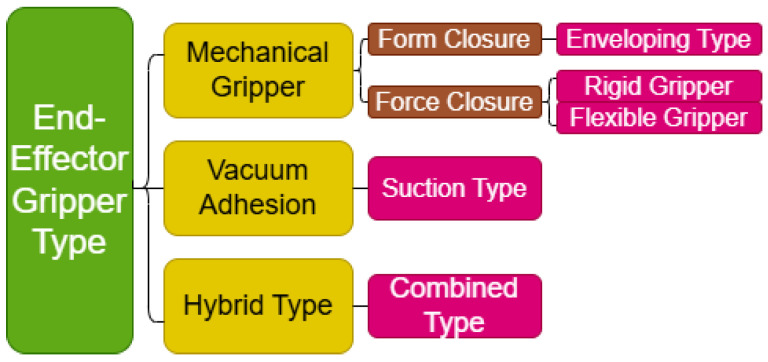
Classification of end effectors based on grasping principle.

Grasping is a key link in harvesting operations to ensure stable fruit posture and prevent dropping. Based on the main physical interaction modes reported in the literature, the grasping mechanism is divided into five engineering categories, which reflect the common design solutions in agricultural harvesting. Including envelope type, rigid gripper, flexible gripper, adsorption type and combined type (such as the form of combining suction and grasping). These categories are not derived from a single classification standard; instead, they reflect the configuration most commonly used in practice. Figure 1 provides a hierarchical view that clarifies the relationship between these categories. These different grasping mechanisms have their own advantages and disadvantages and are suitable for different harvesting scenarios.

#### 2.1.1. Enveloping Type End-Effector

The envelope gripper uses shape closure (such as cage structure) to limit the degree of freedom of the fruit, thereby minimizing the pressure point. They are very stable for spherical fruits such as apples and oranges, but their huge shape limits their use in dense canopy [13].

#### 2.1.2. Rigid Gripper Type End-Effector

The rigid gripper type end-effector is one of the most common grasping devices in agricultural harvesting robots. The rigid gripper is simple and low-cost, but it has the risk of stress concentration and fruit damage. Recent work has improved safety and reduced damage rate by adding force feedback (e.g., Wang et al. [15]) or using rigid flexible coupling (e.g., table grapes [16]).

#### 2.1.3. Flexible Gripper Type End-Effector

The soft polymer gripper adapts to the shape of fruit through hyperelastic deformation [18]. Their damage risk is low, but they face the challenge of durability in the field in ultraviolet and pesticide environments, which is rarely quantified in the current research.

#### 2.1.4. Suction Type End-Effector

The vacuum suction cup can gently grab smooth fruits (apples, tomatoes). On rough or wet surfaces, their reliability will be reduced, and mistakenly sucking of blades is a frequent field problem [19].

#### 2.1.5. Combined End-Effector

The combined end effector (such as the end effector with integrated suction and grasping) first pulls out the fruit from the leaf, and then clamps it. This improves the success rate under occluded conditions (e.g., 95.3% in Chiu et al. [20]), but increases complexity and cost. below summarizes the comparative advantages and limitations of all five types.

Table 1 below summarizes the characteristics of each type.

However, the comparison in Table 1 comes from studies with significant differences in experimental environments. For example, rigid grippers for apples typically operate in low-obstruction structured orchards, while flexible grippers for strawberries are often tested in highly cluttered plastic greenhouses. So this highlights the advantages of combined end effectors in environmental adaptation but also reflects their higher cost. If factors such as occlusion degree, fruit visibility, and branch interference are not controlled, this can only be considered a qualitative comparison.

### 2.2. Detachment Actions

Detachment is the process of removing the fruit from the plant. This action provides the detachment force to break the connection between the crop and the plant. Detachment actions are divided into motion detachment and cutting detachment.

#### 2.2.1. Motion Detachment Methods

Motion detachment refers to the method of detaching the fruit from the plant through physical movements such as twisting, pulling, bending, or suction breaking by the end-effector. Table 2 compares different mechanical fruit-picking methods.

It is important to note that the definition of successful separation varies significantly across different studies: some only require the fruit to be detached from the pedicel, while others mandate the absence of pedicel tearing or similar damage. Meanwhile, studies on mechanical separation rarely evaluate whether the fruit remains commercially viable, and research on cutting-based methods also seldom quantifies blade wear. These inconsistencies undermine the reliability of direct comparisons between the performance of different separation approaches.

#### 2.2.2. Cutting Separation Method

Cutting detachment refers to using tools such as scissors, rotating blades, hot wires, or even lasers to directly cut the fruit stem, as detailed in Table 2. This method works when the stem or stalk is still attached to the fruit. Compared with motion detachment, the cutting mode ensures a clean cut, minimizing physical impact on the fruit and plant. For example, Kondo et al. [61] developed an end-effector for harvesting tomato clusters. This actuator surrounds the main stem of the tomato plant with two upper and lower pairs of finger-like structures. Subsequently, the robotic arm drives the actuator to move along the main stem. When a sensor on the lower finger detects a peduncle, the robotic arm continues moving, bringing the peduncle into contact with a cutter fixed at the upper finger to complete the cut.

In practical applications, the detachment action of the same end-effector is usually a combination of the above motion methods or a combination of motion detachment and cutting detachment. For example, an end-effector mounted on a harvesting system can detach fruits by combining pulling and rotating, pulling and cutting, suction and cutting, or grasping and cutting. However, this method places extremely high demands on visual recognition accuracy. Xiao [41] designed an end-effector integrating pulling and cutting functions. The system leans hard on visual recognition to spot the fruit and stem and get a fix on them.

The design of the gripping and detachment stages needs to take into account the biological characteristics of the fruit. To adapt to the harvesting of a wider variety of fruits, future efforts should focus on developing intelligent end-effectors with rigid-flexible coupling and multimodal fusion, so as to further improve the success rate of picking in unstructured environments and reduce the rate of fruit damage. However, whether for gripping or detachment, the realization of these actions cannot be achieved without the support of the drive system. The drive method directly determines the output torque, response speed, and environmental adaptability of the end-effector. Therefore, this section further provides a systematic review of the drive methods for end-effectors.

### 2.3. Drive Methods of End-Effectors

The drive system is the power source of the end-effector, determining the mechanism’s output torque and response speed, among other factors; while transmission and control strategies directly affect the precision and compliance of the operation. In unstructured agricultural environments, an ideal drive system needs to strike a balance between force control precision, position control precision, and environmental tolerance. According to the differences in energy conversion media, existing drive methods are mainly divided into three categories: electric, fluid, and cable-driven. In addition, new drive technologies based on smart materials are gradually emerging due to their bionic characteristics.

#### 2.3.1. Electric Drive

The electric drive provides mature high-precision control, but the inertia of the end effector is increased due to the weight of the motor [53,62,63,64]. To compensate for the excessive weight of the motor itself, some studies have adopted a hybrid electric and pneumatic drive strategy. For example, Jun et al. [65] designed a highly integrated picking robot. The characteristic of this robot is its ability to decompose tasks, using pneumatic technology for compliant adaptation and electric drive for precise execution.

#### 2.3.2. Fluid Drive: Hydraulic and Pneumatic

Fluid drive can be divided into hydraulic drive and pneumatic drive. Hydraulic drive is difficult to achieve widespread application due to common problems such as excessive oil temperature and low working efficiency. Moreover, when operating in the field, it is prone to liquid leakage, which contaminates crops and soil [66].

In contrast, pneumatic technology, owing to its advantages of high efficiency and reliability, is widely used in agricultural picking. In agricultural robot end-effectors, to address the issues of poor compliance and slow response speed associated with traditional soft grippers, Xie et al. [67] achieved efficient strawberry picking in complex ridge-planting environments through a technical approach combining pneumatic cutting of fruit stems, integrated visual positioning, and dual-arm collaboration. Zhou [68] proposed a rigid-flexible hybrid structure mimicking a human finger, consisting of an outer soft body and an internal rigid skeleton. The soft body protects the fruit, while the skeleton provides fast response. Although pneumatic grippers offer good compliance, they typically suffer from weak load-bearing capacity. Therefore, recent research has introduced structures based on the Fin Ray effect, which have passive adaptability and can wrap around spherical fruits, improving picking success rates without sacrificing compliance [69].

#### 2.3.3. Underactuated and Cable-Driven Mechanisms

Whether electric or fluid-driven, the actuators are usually directly mounted at the end, increasing end inertia. To fundamentally solve this problem, cable-driven technology places the power source at the base and transmits motion and force remotely through cables. Typically driven by an electric motor, this technology addresses the end inertia problem found in underactuated systems—that is, systems where fewer motors control a greater number of joint degrees of freedom. However, the friction loss and nonlinear complexity of control models brought about by long-distance transmission are currently the main technical difficulties [70,71].

#### 2.3.4. Biomimetic Drive Based on Smart Materials

With the advancement of materials science, using smart materials such as Shape Memory Alloys (SMAs), Electroactive Polymers (EAPs), and Ionic Polymer–Metal Composites (IPMCs) as artificial muscles has opened new paths for the miniaturization and integration of end-effectors [72,73].

##### Shape Memory Alloys (SMAs)

An SMA is a special alloy that can recover its original shape after deformation upon heating and can fully recover under certain stimuli. This smart material has an extremely simple structure and is noiseless. However, its thermal response speed is slow, leading to low work efficiency, making it difficult to meet the speed requirements of commercial harvesting [74,75,76].

##### Electroactive Polymers (EAPs)

Electroactive polymers (EAPs) can produce significant deformation under an electric field. They have fast response speed and good flexibility. Based on their unique properties, EAPs show great application potential in many fields, especially in end-effectors for agricultural picking robots [77]. However, most current EAP materials require high driving voltages (kilovolt level) and have small output forces, still in the laboratory exploration stage. Ionic Polymer-Metal Composites (IPMCs), as a subclass of EAP, have shown specific application examples, mainly in flexible grippers with force sensing capabilities. For example, Chattaraj et al. [78] designed a flexible gripper consisting of an active IPMC jaw and a passive PDMS jaw, utilizing the compliance of IPMC. This design calculates the force applied to the object during grasping by measuring the deformation of the passive PDMS jaw, thereby providing crucial force feedback. In summary, in terms of reducing size and accommodating more components in smaller spaces, smart material drive technology indeed has promising prospects.

##### Dielectric Elastomer Actuator (DEA)

In recent years, a dielectric elastomer actuator (DEA) has been developed rapidly in the field of agriculture due to its high energy density, large strain and rapid response [79,80,81,82]. But at present, a DEA in the agricultural field is limited by high driving voltage, the protection ability of flexible electrodes in the field, and aging problems. It is difficult to achieve rapid application and promotion in a short period.

##### Hydrogel

Hydrogel has shown unique potential in the construction of adaptive software drivers and sensor integration units due to its excellent biocompatibility, high water content and reversible response to environmental stimuli [83,84]. But at present, the application of hydrogel in agricultural harvesting end effectors is only in the stage of research and exploration.

**Figure 2:**
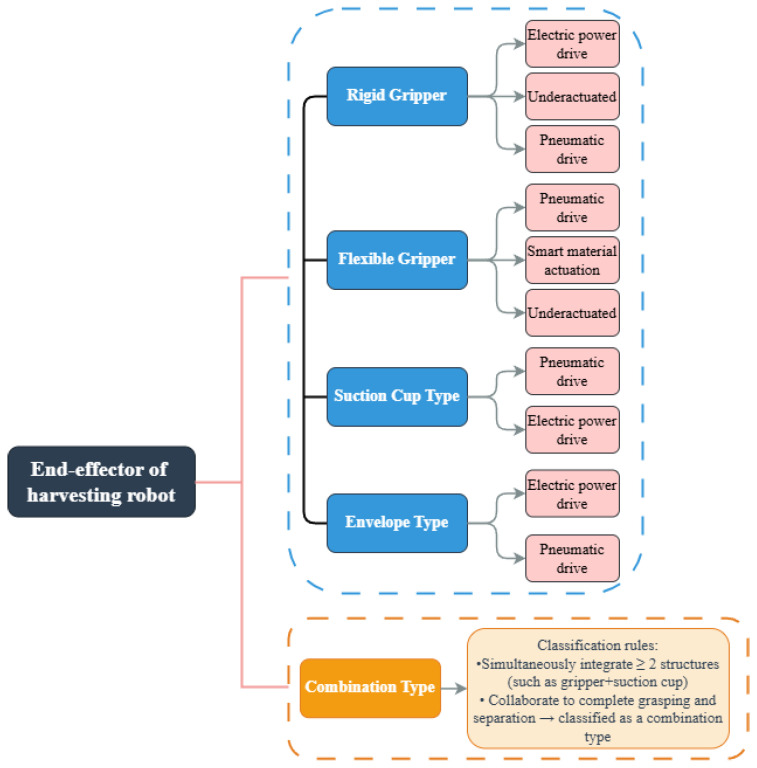
Classification standards and principles of end-effectors.

In order to integrate the two dimensions of structure type (Table 1) and driving principle (Table 3), a unified classification diagram is provided, and the classification criteria and classification rules of combination design are also described, as shown in Figure 2. The summary is shown in Table 3.

In short, there is no single capture or drive method that can adapt to all picking scenarios. The selection should consider the success rate, damage rate and environment. At present, most of the reported performance data come from controlled experiments.

### 2.4. The Importance of Vision, Positioning and Motion Planning for End Effectors

**Figure 3:**
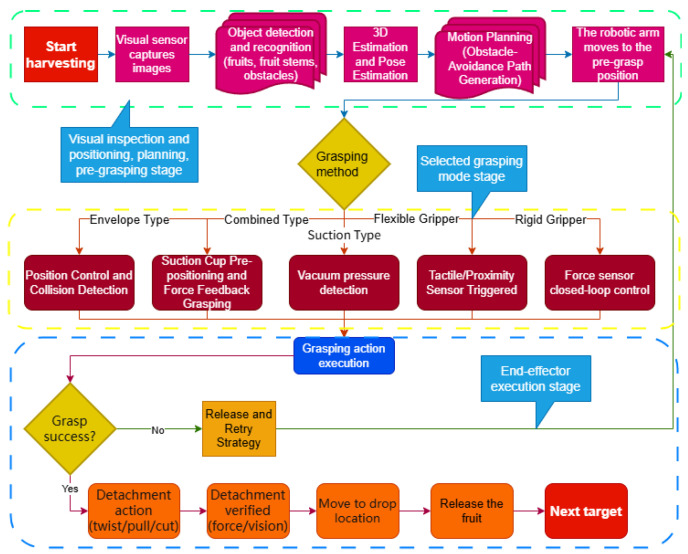
Overall control flow chart of robot picking based on vision.

Although the previous section focuses on mechanical design and drive, recognition, positioning, obstacle avoidance and real-time control are also essential to modern agriculture, as shown in Figure 3. Different types of end effectors put forward different requirements for perception and planning.

Rigid grippers usually require small contact area, high stiffness and high precision positioning. At the same time, they also need precise force control to avoid scratching the fruit. Without accurate 3D pose estimation, the rigid gripper will often miss fruits or cause damage [12]. The flexible gripper can easily produce positioning errors during operation. Usually, in order to ensure that the fruits are not damaged due to excessive grasping, they need touch to trigger and ensure that they are grasped at the appropriate time. At the same time, the precision of visual servo is low and the cycle time is shortened [24,30]. The end effector based on suction is sensitive to occlusion and needs a smooth fruit surface. Therefore, it is necessary to use active vision or multi view planning to verify whether the sucker can contact the fruit without interference from leaves or stems. At the same time, the vacuum pressure must be monitored and adjusted in real time [48,59]. The combined (end effector integrating adsorption and grasping) design has the highest perceptual control coupling. The adsorption phase needs to rely on vision for target capture, while the capture phase needs more refined obstacle avoidance and alignment. This usually requires a stretching and sucking motion, and a positioning fine-tuning phase [49,65,68]. The envelope method has low requirements for the positioning accuracy of fruit, but it highly depends on the perception of obstacles and path planning to avoid hitting branches. Collision detection and re-planning are more important than positioning accuracy [33,44].

In short, there is no single solution for all end effectors. Rigid grippers require high-precision vision and force feedback; flexible grippers rely on tactile perception; suction type requires anti-occlusion vision; the combination type requires mixed planning. Future research should highly integrate the sensing and control capabilities of end effectors.

## 3. Successful Applications of End-Effectors

Agricultural harvesting end-effectors can be classified not only according to operational steps but also subdivided according to the type of crop. Different types of crops have significant differences in geometric dimensions, growth patterns, and tissue fragility, which impose differentiated requirements on the structural design of end-effectors. This chapter analyzes the specific adaptability of existing end-effector solutions and their field operation performance.

### 3.1. Single-Fruit Crop Harvesting End-Effectors

Single fruit crops have a high degree of regularity in shape and are suitable for the use of rigid grippers or composite end effectors. The main bottlenecks are the shading of branches and leaves and the variation in fruit diameter. Apple’s best end effector type is composite, with a success rate of 87% and a damage rate of less than 5%. Although the active obstacle avoidance technology can provide visual guidance for the manipulator according to whether the apple is covered or not, the main reason for its failure is that the branches and leaves are covered [96]. The best choice for tomatos is a flexible or pneumatic gripper, for which the success rate can reach 90%, and the damage rate is less than 5% [21]. The damage rate can be controlled below 5% by using the pneumatic-driven flexible silica gel cavity [37]. The main challenge is that the peel is too thin and easy to damage, and it is difficult to control the mature tomato. Citrus is most suitable for an under-actuated flexible gripper due to its large size difference. The success rate can reach 95.23%, and the damage rate is less than 5%. The challenge also lies in the large fruit diameter difference [40,41]. Kiwifruit picking is the most effective way to use two arm cooperation, and the success rate can reach 86.67% [23]. It is also possible to integrate a composite picking strategy that integrates grabbing, picking and antiskid, with a success rate of 90% and a damage rate of 10% [54], both of which are mainly challenged by the problem of fruit density. Compared with other categories, single fruit crops have the highest requirement for positioning accuracy.

### 3.2. Fruit Cluster Harvesting End-Effectors

The main challenge of fruit cluster crops is to identify fruit stalks and handle multiple fruits at the same time. Occlusion and clustering were the main causes of failure. The best picking method of strawberry is the combination of a flexible gripper and visual recognition. The success rate can reach 62.4% and the damage rate is less than 5%. The main obstacle is cluster shielding [30]. Under the dual arm structure, vision can accurately identify from multiple perspectives, and the success rate after the second test can reach 75–100% [24]. Grapes need compound grippers with active vision [27]. By combining with advanced algorithms, the success rate can reach 87%, and the damage rate is 0.23% lower [97], but the fruit stalk positioning is difficult. Cherry tomato is the most suitable for a textured flexible gripper, the success rate can reach 95.82%, and the damage rate is 2.9% [53], But it is easy to fail because the fruit stalk is flexible [35]. Compared with other categories, clustered crops are highly dependent on visual detection of fruit stalks.

### 3.3. Elongated and Hard-Pedicel Crops

**Figure 4:**
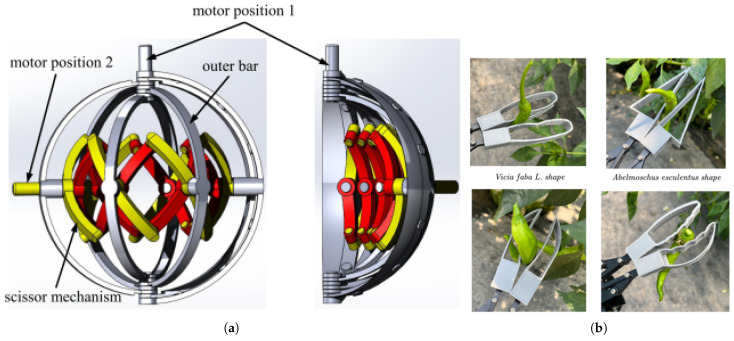
Picking mechanism suitable for peppers: (**a**) Scissor mechanism end-effector [12]. (**b**) Flexible end-effectors of various shapes [17] (open access).

The difficulty of crops with long stalks or hard stalks is that the shape of the fruit is irregular and the fruit stalks are hard and difficult to break directly, so they usually need to be cut. The success rate varies greatly with the accessibility of the causal handle. The best choice for sweet pepper is rigid scissors or a flexible profiling gripper, the success rate can reach 95%, and the damage rate is less than 1.7%, but the irregular shape is still a challenge [12,17]. The specific details are shown in Figure 4a,b. The most effective picking method for cucumber is a suction cup and cutting compound clamping claw, with a success rate of 56.6% and a damage rate of 4.7%. The main problems are that the fruit stalk is blocked and the period is too long [49]. Pumpkin needs a rigid five finger bionic gripper to cooperate with cutting, and the success rate is 79% to 92%, but the damage rate is 21%, which is mainly limited by the fruit weight and vine interference [55]. Eggplant benefits from the cutting and grabbing of two-arm cooperation, with a success rate of 91.67% and a damage rate of less than 5%. The key bottleneck is the toughness of the fruit stalk [36]. Compared with other categories, these kinds of crops most often need an integrated cutting mechanism.

Rigid cage-type mechanisms perform better in terms of reliability and preventing drops, making them suitable for varieties with hard stems; while flexible fit solutions show irreplaceable advantages in handling highly curved or deformed fruits. This also provides a basis for selecting end-effectors for different varieties of sweet peppers in the future. All compiled data are shown in Table 4 for comparison.

In all the studies included in this table, the following engineering parameters have never been reported: contact stiffness/elastic modulus of clamping surface, weight of end effector, energy consumption per pickup, service life of consumables (blades, suction cups, flexible fingers), and replacement frequency or quantitative maintenance requirements. Therefore, a direct engineering comparison based on these indicators is currently impossible. This gap is not the limitation of our review, but the reflection on the existing literature.

The data in Table 4 are from the environment, with great differences in experimental conditions. Laboratory tests usually refer to the appearance of a single fruit without shading, while greenhouse tests may include some branches and leaves but the light is controllable, while field tests often involve complete shading, natural wind and uneven light. At the same time, the evaluation methods of success rate are not unified, so the success rates can not be directly compared. The assessment methods of damage rate are also not uniform, ranging from visible bruises to microscopic epidermal abrasions. Therefore, the values in the table are only regarded as indicators under specific research conditions.

**Figure 5:**
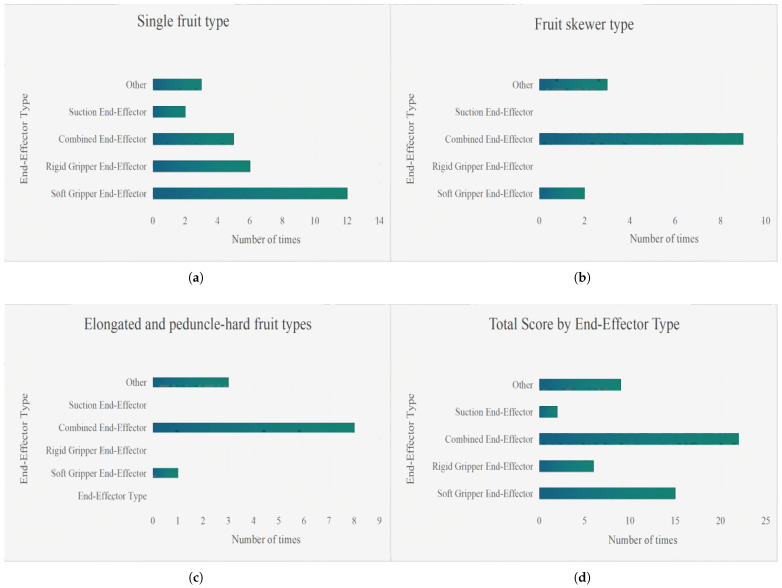
Application statistics of different end-effectors for various fruit types: (**a**) Single fruit type. (**b**) Fruit skewer type. (**c**) Elongated and peduncle-hard fruit types. (**d**) Total number of different end-effectors.

To further elaborate, Figure 5 categorizes and statistically analyzes the suitability of various end effectors from the perspective of fruit morphology.

As shown in Figure 5a–d, combination-type end-effectors have already outpaced all other types put together in terms of total adoption. Their broad adaptability—handling everything from single fruits to clustered crops and those with long, stiff stems—plus their inherent stiffness advantage makes them the clear direction the field is heading in. As a result, research is now shifting heavily toward smarter, multi-modal integrated designs.

**Figure 6:**
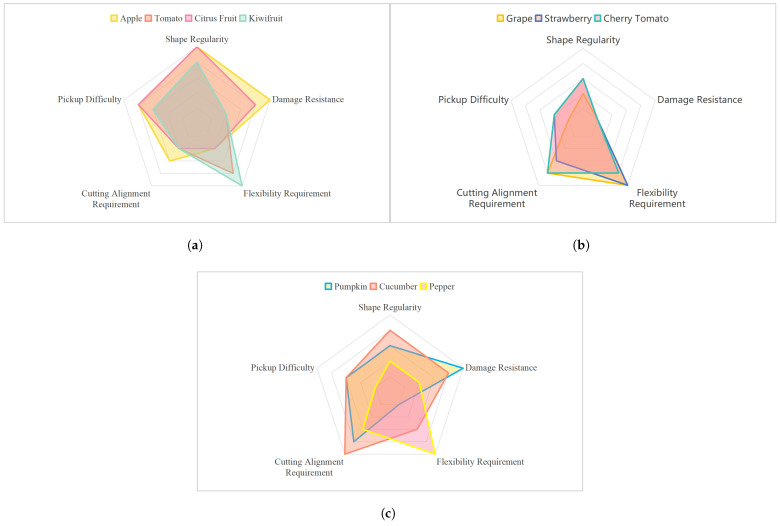
Morphological classification of fruits and vegetables for end-effector design: (**a**) Spherical or near-spherical crops. (**b**) Soft-skinned or cluster-growing crops. (**c**) Elongated or large-mass crops.

To further understand why different fruit types require different end-effector configurations, we next analyze the intrinsic physical properties of these fruits. Figure 6 presents radar charts that qualitatively compare representative crops across five key characteristics derived from literature-reported data.

Based on the radar charts shown in Figure 6a–c, the scores (1–5) are ratings assessed by the author and derived from a comprehensive evaluation of the physical properties reported in the literature, as well as the observed difficulty in robotic handling. A score of 5 indicates the most prominent requirement or the highest challenge for an end-effector when dealing with a given fruit type.

As can be seen in Figure 6a, apples show high scores in all dimensions, indicating the best general adaptability to mechanical grasping and handling. Overall scores for tomatoes, citrus and kiwifruit decrease in sequence, with the kiwifruit scoring the lowest in the resistance dimension to damage, consistent with its soft flesh and susceptibility to damage. Additionally, all products score high in the shape regularity dimension, reflecting that single fruits are generally geometrically regular and easy to position.

Figure 6b compares the differences between two cluster-type agricultural products (strawberry and cherry tomato) across three dimensions: damage resistance, grasping flexibility, and shape regularity. Strawberries have stronger damage resistance, while cherry tomatoes are more regular in shape but have higher picking difficulty. This suggests that customized designs are needed for different fruit clusters.

Figure 6c shows the differences in characteristics among three irregularly shaped products: pumpkin, cucumber, and bell pepper. Pumpkin has the highest demand for compression/damage resistance but the lowest demand for flexible conformability, making it suitable for rigid clamping. Cucumber has a high demand for peduncle/cutting alignment, which is consistent with its characteristic of having an obvious peduncle that is often hidden and requires precise positioning. Bell pepper has the highest demand for flexible conformability and the lowest demand for grasping/contact difficulty, reflecting the slippage problem caused by its smooth surface and high-curvature shape, which necessitates flexible adaptive clamping. The results indicate that for irregular agricultural products, gripper design needs to specifically address issues of slippage, positioning, and morphological adaptation.

In summary, the statistical counts in Figure 5 and Figure 6 show good consistency with the performance data in Table 4, indicating that the selection of end-effectors requires comprehensive consideration of the matching relationship between fruit morphology and drive method. Among them, flexible grippers have obvious advantages for single-type crops, while combined and rigid grippers show higher application potential for cluster-type and long-pedicel, hard-stem crops, respectively. With the development of artificial intelligence and machine vision, the future end-effector will no longer be a purely mechanical gripper but an intelligent system integrating perception, decision-making, and execution.

### 3.4. Derivation of Ten Representative Crop End Effector Combinations

The results showed that the design of the end effector was closely related to the biological characteristics of crops. The ten combinations listed in Table 5 below are not exhaustive design guidelines, but only represent the pairing of the most frequently reported and performance verification in the literature (2010–2025). They were mainly selected through the following two steps:

1. Selection criteria: In the literature, at least three independent studies have reported the quantitative performance (success rate, damage rate and cycle time) of end effector types. In terms of fruit morphology, the combination belongs to the spherical single fruit (such as apple, citrus, tomato, etc.), cluster fruit (such as strawberry, cherry tomato, etc.) or slender and hard stem fruit (such as pepper, cucumber, eggplant, etc.) determined in Figure 6. In terms of data, the quantitative data in Table 4 (success rate, damage rate, picking time) and the qualitative assessment in Figure 6 are applicable to crops. At the same time, less than three studies have reported complete indicators, and the reported success rate or failure rate is far lower than the listed combination, so they are excluded.
2. Deep connection: For example, strawberries require high grip flexibility and are vulnerable to injury, which requires the selection of a flexible gripper (highly flexible and flexible end effector). Therefore, Figure 6 explains why a given end effector type is appropriate, while Table 4 provides actual performance evidence.

**Figure 7:**
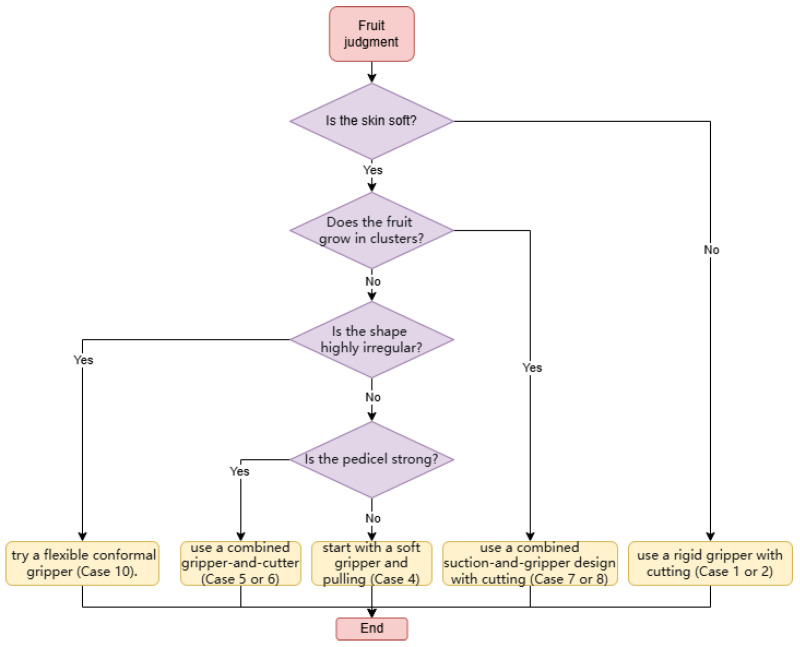
Simplified process reference diagram.

This table can serve as a basic reference for end-effector design. A simplified flow chart is shown in Figure 7.

Nevertheless, various design methods for end effectors have been discussed earlier, and there are still many challenges when working in unstructured environments. These challenges include aspects such as biological heterogeneity, perception limitations, energy efficiency, material durability, and environmental adaptability.

## 4. Key Technical Bottlenecks and Future Outlook in Unstructured Environments

Although significant progress has been made in the above-mentioned operational mechanisms, drive technologies, and practical applications, existing agricultural harvesting robots still lag significantly behind skilled human labor in terms of operational efficiency, environmental adaptability, and robustness. The root of this gap lies in the contradiction between the highly unstructured agricultural environment and the limited perception and execution capabilities of current robots.

To deeply analyze these contradictions, this chapter first systematically sorts out the core challenges in end-effector design from six dimensions: adaptation to biological heterogeneity, flexible perception and feedback, the trade-off among efficiency, precision, and damage rate, energy endurance, material durability, and the limitations of smart materials. On this basis, to further enhance environmental adaptability and robustness and bridge the gap between laboratory prototypes and real field applications, this chapter proposes a Field Deployability Checklist. This checklist defines quantitative evaluation indicators from five engineering usability dimensions: dustproof/waterproof, easy cleaning, easy maintenance, anti-aging, and aspiration prevention, aiming to guide researchers in reporting quantitative results of these engineering indicators alongside grasping performance. Finally, based on quantitative statistics of the usage frequency of various end-effectors, the current application status and structural characteristics of the technology are summarized, and future key research directions are prospected accordingly.

### 4.1. Biodiversity of Target Objects and Generalized Adaptability

The diversification and non-standardization of crops, along with the discrete distribution of their morphological parameters, pose great challenges to the universal design of end-effectors.

Regarding the uncertainty of biological morphology and scale: For the same fruit variety during the ripening period, the diameter variation can exceed 30%, and various shape manifestations such as spherical, oblate, and malformed exist. This requires the end-effector to achieve effective envelopment of the target object through its own underactuated characteristics or large opening stroke, even in the absence of precise 3D model matching [98,99].

Regarding the complexity of the crop growth environment: Vine crops often exhibit random twining growth, and fruits are frequently hidden deep within foliage or clustered together. In this scenario, Wu et al. [100] designed a multi-degree-of-freedom adaptive finger with joint angle measurement capability, which effectively improves the dexterity of the gripper. Meanwhile, actuators with bendable or extendable structures can also be designed to adapt to different growth environments [101,102]. Currently, almost all data comes from controlled laboratory environments or greenhouses. Only a small portion of research is conducted in field environments. This inevitably leads to a lower success rate for actual harvesting in the field.

### 4.2. Flexible Interaction, Tactile Perception, and Feedback

When designing end-effectors for agricultural harvesting, accuracy and flexibility are two crucial factors. As long as these two aspects are well addressed, crop damage can be effectively reduced, and harvesting efficiency can be significantly improved.

Regarding the biological characteristics of fruits, the firmness of the fruit skin is not a constant value but changes dynamically with ripeness. This requires the force sensor to precisely control the gripping force so as to reduce fruit damage. Pressure sensors have been integrated into flexible fingers for real-time grasping force monitoring [44]. This stabilized the grasping force within a range that ensures firm grasping of the fruit without causing damage.

Although force feedback can effectively control the contact force, in actual field environments, fruits are often occluded by branches and leaves or under backlighting conditions, causing the vision system to fail to accurately identify the target. When vision fails, relying solely on force feedback also makes it difficult to complete grasping. Therefore, multimodal perception technology integrating touch and vision is gradually becoming a solution. Research shows that such methods can effectively handle visual blind spots [103].

Most force sensors are non-standard products and are difficult to generalize. Vision can only provide partial damage control, but multimodal technology with force sensors can better reduce fruit damage. Therefore, it is necessary to implement standards uniformly.

In addition to the control strategy, the force sensing (control) ability greatly affects the performance and actual reliability of the end effector. Table 6 (from [83,104]) compares six common sensing modes. The piezoresistive type is widely used because of its flexibility and low cost, but its long-term stability and aging problems are prominent in the open environment. The capacitive type has high sensitivity but is easily disturbed by the environment (humidity, etc.), and thus is only used extensively in greenhouses. The piezoelectric type has a fast dynamic response, but it is difficult to measure the static gripping force, so it can only be applied to the monitoring of the instant impact of gripping. Fiber Bragg grating (FBG) has high precision, but it is expensive and difficult to integrate, so it is difficult to commercialize and apply. Therefore, in order to improve the force perception ability, using multi-mode fusion (such as piezoresistive and capacitive joint measurement of static and dynamic forces, etc.) combined with the current advanced algorithm is the best way at present.

### 4.3. The Ternary Trade-Off Among Efficiency, Precision, and Damage Rate

It is important to balance the damage rate, operation speed and picking accuracy. In order to optimize this problem, the current methods use artificial intelligence and machine learning to analyze a large number of harvest data, so that the system can continuously optimize the control strategy to adapt to different harvest scenarios and fruit characteristics [105]. However, most of the existing studies evaluate the performance of end effectors under quasi-static conditions or low operating speed. Therefore, in order to match the human picking efficiency, the manipulator must move at a higher speed, which will introduce residual vibration in the end effector. These vibrations will reduce the positioning accuracy and increase the risk of damage. At present, only a few studies have made quantitative analysis of this dynamic effect, and no real-time compensation strategy has been proposed. This is the premise of commercial deployment.

### 4.4. Energy Self-Sufficiency and Lightweight Design

Energy efficiency is a critical bottleneck that urgently needs to be addressed in the agricultural application of end-effectors. Unlike fixed robotic arms in factories that are powered by the grid, mobile harvesting robots rely entirely on their own batteries.

Regarding energy consumption, as the most frequently moving component, the mass of the end-effector directly determines the power consumption of the robotic arm’s joint motors. Heavy end-effectors not only reduce the robot’s operational runtime but also increase the system’s rotational inertia, thereby degrading dynamic response and compromising harvesting accuracy.

In terms of design optimization, lightweight design is of paramount importance. This involves not only the use of low-density materials such as carbon fiber and engineering plastics, but also the elimination of redundant structures through topological optimization. Currently, developing drive circuits with energy recovery capabilities or utilizing energy storage components such as springs to balance gravitational potential energy are effective ways to reduce overall energy consumption. Electromagnetic induction-based energy recovery has been proposed to extend battery life [85,106,107].

### 4.5. Material Durability and Maintenance Cost

The end effector must maintain stable performance in the field environment [108,109].

Regarding material aging, especially for silicone, rubber, and smart materials widely used in flexible grippers, they are highly susceptible to aging, hardening, or fatigue fracture under long-term outdoor exposure, which is one of the main challenges currently faced. To further optimize friction and wear between cutting tools, Panda et al. [110] developed a polyaryletherketone composite material. Due to its low friction coefficient and very little wear debris, it is suitable for use as a surface coating for cutting tools. Cooke et al. [111] developed a cutter blade based on a novel coating material. This coating improves the tribological performance of the cutting tool and significantly extends the cutting life of sugarcane harvester blades.

Although many studies have emphasized the advantages of soft and flexible grippers, few reports have conducted systematic accelerated aging tests (UV, temperature cycling, pesticide exposure). Without these data, the long-term performance of flexible end effectors in real environments is still unknown. This is also a core aspect of the performance indicators listed in Table 6.

Regarding maintainability, the end-effector should be a modular component that is easy to clean, disinfect, and can be quickly replaced in case of failure, rather than a precision instrument. For some key moving parts such as gears and joints, wear-resistant and fatigue-resistant materials are needed to withstand repetitive motion, effectively ensuring the long-term precision stability of the robotic arm’s operation [112].

### 4.6. Environmental Limitations of Smart Materials

The driving core of SMA lies in thermally induced phase transformation, with its phase transformation temperature typically designed between 40 °C and 90 °C. Due to the large temperature span in agricultural environments, at low temperatures, SMA requires increased heating power to achieve full contraction, while at high temperatures, slow heat dissipation leads to prolonged cooling times, making continuous operation difficult. Meanwhile, during field operations, strong winds can change the convective heat transfer coefficient on the SMA surface. Even with a constant current, the temperature rise curve of SMA becomes difficult to predict, leading to unstable control of the end-effector.

The vast majority of high-performance EAPs rely on kilovolt-level high voltages to generate electrostatic driving forces. In agricultural field environments, high humidity can easily create risks of electrical leakage; high dust levels can cause dust to adhere to the electrode surfaces of EAPs, resulting in local electric field distortion. This makes voltages that are safe in a cleanroom environment no longer safe in the field.

### 4.7. Lack of Engineering Parameters

Although Table 4 provides quantitative data on harvesting success rate, damage rate, and cycle time, actual engineering parameters are scarce in existing literature (2010–2025). This section is based on a systematic search of the literature and evaluates the reporting status of these parameters one by one.

For the stiffness or elastic modulus of contact elements, a few studies reported the contact stress (MPA) or material hardness (Shore A). For example, Xie et al. [18] reduced the maximum contact stress of flexible fingers by about 70% through topology optimization, and gave the damage stress threshold of apple (0.13 MPa), strawberry (0.093 MPa) and other fruits; Kargar & berselli [113] tested the friction behavior of silica gel cushions with 10a and 30A shore hardness under different textures, loads and speeds. However, the elastic modulus of flexible fingers lacks a unified characterization method among various studies, and most literature only qualitatively mention the material type and do not give a comparable stiffness value.

For the weight of the end effector, the weight parameters are only reported in individual studies. The soft cable-driven apple picking gripper developed by ninatanta et al. [114] weighs 0.306 kg; the two kinds of flexible grippers designed by Xie et al. [18] weigh 87.43 g and 84.41 g, respectively. The design mass of a tomato picking end effector reported by Wang et al. [52] is about 1.2 kg. However, most studies have not reported the weight of the end effector at all, which makes the horizontal comparison based on weight index impossible.

For the energy consumption of a single operation, none of the existing literature has reported the energy consumption of a single picking (J/pick). Bras et al. [115] reported the power consumption of an agricultural robot (the instantaneous power is up to 12 kW, and the average energy consumption is 250 WH/task), but this is the total energy consumption of continuous operation, which cannot be decomposed into a single harvest. The accurate measurement of single harvest energy consumption is blank in all literature related to agricultural harvesting end effectors.

Flexible grippers, suction cups, blades and other vulnerable parts have a short life in orchard environments. Șerdean et al. [116] predicted the theoretical life of FESTO pneumatic muscle (about 994,482 contractions) based on Weibull distribution and Monte Carlo simulation, but the study was based on generated data rather than long-term field measurements. Ninatanta et al. [114] reported that its soft gripper prototype can be maintained within 40 picking cycles without maintenance, but did not give longer durability data. No research system has reported the service life of the flexible gripper under real orchard conditions.

For the replacement frequency of suction cups, blades and flexible components, the quantitative replacement frequency of suction cups, blades and flexible components is completely missing in the field of agricultural picking robots. The preventive replacement model proposed by Șerdean et al. [116] recommends replacement at 984,000 contractions (about 164 days), but this is still based on the estimation of modeling rather than on-site empirical data.

For maintenance requirements, most of them are qualitative descriptions. The tomato picking end actuator designed by Wang et al. [52] is maintenance free (no lubrication). Șerdean et al. [116] proposed a maintenance cost minimization strategy based on the failure probability threshold, but did not give a specific maintenance cycle or cost value. The review by vrochidou et al. [117] only outlines the low maintenance requirements. Data that quantify maintenance cycles and maintenance costs are not reported in all literatures.

The lack of the above parameters is not a criticism of a single study, but shows that many studies focus on the proof of concept or controlled environment verification, ignoring the display, which seriously restricts the engineering comparability and commercialization process.

### 4.8. Field Deployability Checklist

Existing research mostly focuses on grasping and detachment performance, lacking analysis of engineering issues such as dustproof/waterproof, cleanability, consumable replacement, material aging, and aspiration prevention. To address this, this paper proposes a Field Deployability Checklist, taking dustproof/waterproof, easy cleaning, easy maintenance, anti-aging, and aspiration prevention as five core dimensions for evaluating the engineering usability of end-effectors to supplement the existing performance indicator system.

Dustproof/Waterproof is the foundation for field deployment. Agricultural picking robots often operate in environments with dew, dust, branch and leaf friction, and washing. Motors, sensors, and electrical connectors in the end-effector are prone to failure due to water ingress or dust accumulation. Therefore, structural design needs to balance sealing requirements for greenhouse and open field environments.

Easy Cleanability directly affects the repeated use and hygienic safety of the end-effector. Therefore, fruit-contacting parts should be designed as detachable and washable structures, with surfaces as smooth as possible, reducing grooves, gaps, and cleaning dead corners. For example, for flexible conformal end-effectors [17], besides the grasping success rate, attention should also be paid to the surface integrity and frictional stability of the flexible material after multiple contamination-cleaning cycles.

Ease of maintenance directly affects work efficiency and cost. During picking, parts like suction cups and blades can be complicated to replace. To make the system better suited for varying field conditions, a modular design is the way to go. On an end-effector [49] that handles both suction and cutting, the blades and suction cups are wear items—they are meant to be serviced and swapped out periodically. Building the assembly in modules makes that kind of maintenance far more practical.

Mis-suction protection is a key step for suction-type actuators to achieve picking. In environments with dense branches and leaves, mis-suction easily occurs, requiring the suction-type end effector to correctly identify and locate targets while being equipped with mis-suction detection. For crops such as cucumbers, where the space for stem positioning is limited and the branches and leaves are dense [49], the success rate often depends on the accuracy of visual recognition and the fault tolerance during the suction stage.

#### Derivation and Intended Use of Field Deployment Capability Checklist

Promote the development of relevant engineering standards through a comprehensive review of site failures, environmental restrictions or practical issues (such as water ingress, dust accumulation, suction cup blockage, mechanical wear and material aging, etc.) mentioned in all relevant documents. Its advantages are that, on the one hand, the checklist can record important engineering indicators that are often ignored in future research, and on the other hand, researchers can score the existing end effectors (high/medium/low) in different dimensions to evaluate the site preparation of different designs.

This checklist is not a general design standard or maintenance manual. In fact, it is a structured analytical framework to highlight underreported engineering indicators that will limit field performance, as shown in Table 7.

There are few or no quantitative data about dust/waterproof grade, cleaning cycle, maintenance time or anti-aging tests reported in the existing studies. A few are also limited to laboratory accelerated aging tests and lack of on-site verification. This question directly explains why the deployment in the field will fail. Therefore, the checklist can be used not only as a design guide, but also as a diagnostic tool to fill the gap between laboratory and field deployment.

## 5. Summary and Outlook

This review systematically summarizes the research status of the end effectors of agricultural harvesting robots, makes an in-depth analysis from four aspects: grasping mechanism, driving mode, typical crop applications and engineering challenges, and introduces a field deployability checklist that is different from the existing reviews. This chapter quantitatively summarizes the existing technology layout, and looks at future development directions.

### 5.1. Quantitative Summary of Research Status

**Figure 8:**
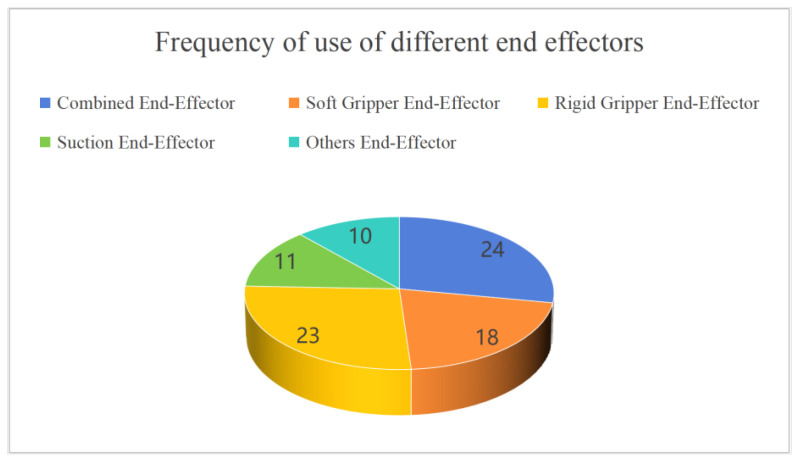
Number of occurrences (frequency) of different end-effectors for grasping actions in the reviewed literature.

The existing end effector designs are highly specific. However, these conclusions mainly come from the qualitative description of specific cases and lack of quantitative analysis of the overall landscape of existing studies. In order to solve this problem, this paper statistically classifies the end effectors that appear in the relevant literature, and the results are shown in Figure 8.

However, the frequency distribution in Figure 8 does not represent the current research direction. The current research progress may be more inclined to the novel modular or flexible design than the rigid gripper with simple structure, mature technology and low cost. In addition, research efforts are concentrated on a few high-value crops (such as apples, tomatoes and citrus), while many important regional crops still lack research. Therefore, the position of combined end effectors in practical applications may not only verify the technical superiority, but also reflect the research trend.

### 5.2. Technology Outlook and Future Development Directions

Based on the statistical analysis of the above charts, the development of end-effectors can be pursued in the following directions.

1. Multimodal sensing: Current end-effectors usually rely on machine vision alone. Future research should first enable the end-effector to identify fruits through vision, and then sense fruit firmness through touch. Recent studies show that agricultural picking end-effectors integrated with multimodal sensing can enhance the system’s adaptability to complex environments and improve the robustness and success rate of picking [117].
2. Variable stiffness actuators: A single rigid or flexible structure struggles to balance load capacity with a low damage rate. Developing end-effectors with variable stiffness characteristics will be a breakthrough point. For example, one study proposed an electromagnetically driven end-effector with adjustable stiffness and coordinated force–stiffness control. Its core innovation lies in decoupling and then synergizing force control and stiffness control. This method provides an effective solution for picking robots to adapt to the differences in stiffness of fruits at different maturity levels and achieve non-destructive clamping [118].
3. Edge computing and deep reinforcement learning: To address the high biological heterogeneity of the operating objects, the end-effector should not only be an actuator but also possess a certain level of edge computing capability. By combining deep reinforcement learning algorithms, the end-effector can continuously and autonomously learn the optimal grasping strategy through physical interaction with the environment, thereby achieving active cognition. The latest deep reinforcement learning algorithms have been applied in practice, equipping robotic hands with the ability for adaptive strategy learning in unknown environments and the capacity to balance inter-regional path optimization with intra-regional interference minimization [63].

## Figures and Tables

**Table 1:** Comparative data of grasping mechanisms.

Gripper Type	Characteristics	Advantages	Disadvantages	Target Crops	References
Rigid Gripper End-Effector	Multi-finger design; grasping force control; modular structure.	Strong adaptability; precise control; customizable.	Limitations with soft crops; complex control system.	Apple; tomato; citrus; strawberries, etc.	[15,22,23,25,28,32,33,34,35,36]
Flexible Gripper End-Effector	High compliance; reduced damage risk; strong adaptive; ability for multiple drive modes.	High safety; flexible design; simplified control.	Limited load capacity; durability issues; manufacturing complexity.	Strawberry; pear; cherry tomatoes, etc.	[17,18,37,38,39,40,41,42,43,44,45]
Suction End-Effector	Vacuum suction principle; flexible suction cup design; strong adaptability to smooth-surfaced crops; low mechanical contact.	Reduced crop damage; simple operation; good adaptability.	Poor adaptability to rough surfaces; relatively high energy consumption; environmental limitations.	Apple; citrus; tomato.	[19,46,47,48]
Gripping-and-Suction Combined End-Effector	Diverse structural designs; multiple drive modes.	Strong adaptability; high picking efficiency; protects fruit shape.	Complex structure; relatively high energy consumption.	Apple; tomato; pear.	[49]
Envelope Type End-Effector	Adjustable structure; conforms to crop shape for enveloping.	Stable grasping, prevents dropping; reduces target damage; compact and efficient structure; adaptive grasping adjustment.	Complex drive structure; difficult to meet high precision requirements; relatively low grasping efficiency.	Strawberry; tomato; apple.	[24,26,50,51,52]

**Table 2:** Comparison of detachment types for agricultural harvesting end-effectors.

Detachment Type	Schematic	Advantages	Challenges	Common Crops	References
Motion Detachment (Natural separation point)	Rotating	Suitable for crops with relatively strong stems.	For crops with fragile stems or those difficult to rotate, may increase the risk of fruit damage.	Apples, Citrus.	[14,19,32,41,43,44,53]
	Pulling	Can be achieved via grippers or vacuum suction; simple control strategy; fast cycle time.	Risk of detachment failure due to insufficient suction force; potential to damage the whole plant or roots.	Certain types of vegetables, flowers or cucumber.	[17,19,21,28,30,31,32,36,46,48,49,53,54]
	Bending	Flexible operation, suitable for plants with softer branches or lightweight crops.	Bending action requires precise control; excessive force may damage the crop or branches.	Strawberries.	[23,25]
Cutting Detachment (No natural separation point)	Knife shearing	Strong adaptability; high precision; widely applicable.	Complex control requirements; maintenance and wear issues; potentially high energy consumption.	Fruit picking; flower picking; root crops.	[12,22,26,27,33,34,35,41,49,55,56,57]
	Heat shearing	High safety; prevents virus transmission.	Significant material wear issues; slow cutting speed.	Root crops.	[58,59,60]

**Table 3:** Comparison of different actuation methods for end-effectors.

Actuation Method	Specific Method	Advantages	Disadvantages	Typical Applications	References
Electric Actuation	Electric Motor (Rigid)	High precision; clean energy; no leakage risk; mature technology; easy to integrate.	High end-effector inertia; rigid contact risks damaging soft fruit; complex force control algorithms required.	Apples; Citrus; Tomatoes (Structured environments)	[12,15,17,18,27,29,34,47,54,55,56,85]
Electric Actuation	Cable-Driven (Underactuated)	Decoupled mass; low inertia; flexible transmission; adaptive grasping; compact end-effector design.	The wire rope transmission system has friction and delays; the wire rope wears quickly and has fatigue problems.	Anthropomorphic hands; lightweight arms.	[70,71,86,87]
Fluid Actuation	Pneumatic Drive (Soft)	High power-to-weight ratio; inherent compliance; fast response speed; low cost; easy to manufacture.	Requires a large-volume air compressor; highly nonlinear; difficult to control precisely; long pipelines can easily lead to efficiency loss.	Strawberries; berries; delicate crops.	[18,42,44,45,46,48,49,52,53,88]
Fluid Actuation	Hydraulic Drive	Extremely high force output; robust against external disturbances; self-lubricating system.	Heavy system weight; risk of oil leakage; slow response speed compared to pneumatics.	Sugar cane; heavy-duty harvesting.	[67,89,90,91,92]
Smart Material Actuation	Shape Memory Alloys (SMAs)	Silent operation; no motor noise; high energy density per volume; simple structure.	Slow response; low energy efficiency; sensitive to ambient temperature/wind.	Experimental micro-grippers; auxiliary actuation.	[74,75,76,93]
	Dielectric Elastomers (DEAs/EAPs)	Fast response; large strain capability; lightweight; soft contact.	Requires high voltage; sensitive to humidity and dust; low blocking force.	Lab prototypes; soft tactile sensors.	[79,80,81,82,94,95]
	Hydrogel	Excellent biocompatibility and high water content.	Slow response speed; small output force; low mechanical strength.	Lab prototypes.	[84]

**Table 4:** Comparison of end-effectors for different crop types.

Crop Type	Picking Scenario	Driving Mode	Successful Rate	Damage Rate	Picking Time	References
Apple	Laboratory test	Electrical	72%	/	14.6 s	[38]
	Field experiment	Electrical	71.28–80.45%	<8%	5.8 s–6.7 s	[64]
	Field experiment	Pneumatic	Young orchard: 82.4% Dense orchard: 65.2%	/	6.0 s	[48]
	Field experiment	Electrical	76.97%	5.56%	7.29 s	[63]
	Field experiment	Electrical and Pneumatic	87%	<5%	/	[19]
Tomato	Greenhouse test	Electrical	>80%	/	9.5 s–18.4 s	[37]
	Greenhouse test	Electrical	90%	<5%	7.5 s	[21]
	Greenhouse test	Electrical	>95%	/	4.65 s	[15]
	Field experiment	Pneumatic	92%	0%	74.6 s	[20]
	Greenhouse test	Pneumatic	83.9%	/	24 s	[50]
	Greenhouse test	Electrical and pneumatic	50%	/	15 s	[61]
Citrus and orange	Laboratory test	Electrical	95.23%	<5%	4.65 s	[41]
Strawberry	Field experiment	Electrical	First experiment: 50–97.1% Second experiment: 75–100%	<5%	Single-arm: 4.6 s Dual-arm: 6.1 s	[24]
	Field experiment	Electrical	53.5%	<5%	7.5 s–10.6 s	[26]
	Field experiment	Electrical	62.4%	<5%	6.8 s–7.6 s	[30]
Kiwifruit	Field experiment	Electrical	82.93%	8.38%	2.3 s–2.5 s	[63]
	Laboratory test	Electrical	90%	10%	4.0 s	[54]
Cherry tomato	Greenhouse test	Electrical	69.4–84%	1.9%	6.4 s	[25]
	Field experiment	Electrical	95.82%	2.90%	4.86 s	[53]
	Greenhouse test	Electrical	57.7%	/	20.86 s	[35]
Grape	Field experiment	Electrical	87%	0.23%	9 s	[16]
Pepper	Greenhouse test	Electrical	83.3–100%	1.7%	/	[17]
Eggplant	Laboratory test	Electrical	91.67%	<5%	26.0 s	[36]
Pumpkin	Laboratory test	Electrical	92%	21%	/	[55]
Cucumber	Greenhouse test	Electrical and Pneumatic	56.6%	4.7%	56.0 s	[49]

**Table 5:** Recommended end-effector configurations (rough estimation based on the reviewed literature).

Property	Case 1	Case 2	Case 3	Case 4	Case 5	Case 6	Case 7	Case 8	Case 9	Case 10
Skin	Hard	Hard	Medium	Soft	Soft	Soft	Medium	Soft	Hard	Soft
Shape	Regular	Regular	Regular	Regular	Regular	Irregular	Regular	Regular	Irregular	Irregular
Cluster	Single	Single	Single	Single	Single	Single	Cluster	Cluster	Single	Single
Pedicel	Weak	Strong	Medium	Weak	Medium	Strong	Weak	Weak	Strong	Medium
Proposed gripper	Rigid gripper	Rigid and cutter	Enveloping or soft gripper	Soft gripper	Combined (suction and gripper)	Combined (gripper and cutter)	Combined (suction and gripper)	Soft gripper and suction	Rigid and cutter	Flexible gripper (conformal)
Separation mode	Pull/twist	Shear cut	Twist and pull	Pull	Suction and pull	Cut and grasp	Suction and cut	Pull or cut	Cut	Pull and twist
Actuation	Electric	Electric or pneumatic	Electric	Pneumatic	Electric and pneumatic	Electric or pneumatic	Pneumatic and electric	Pneumatic	Hydraulic or electric	Pneumatic
Expected success	High	Fairly high	Fairly high	Fairly high	High	Fairly high	Fairly high	Fairly high	Fairly high	Medium
Expected damage	Very low	Very low	Very low	Very low	Low	Low	Very low	Low	Low	Medium

Note: The evaluation conditions of success rate and damage rate in this table are qualitative induction based on the experimental results of the literature reviewed. Success rate rating: high > 85%, fairly high 80–85%, medium 70–80%; damage rate rating: very low < 5%, low 5–8%, medium 8–15%. This table is intended to provide a preliminary reference for the selection of end effectors under different combinations of fruit characteristics.

**Table 6:** Comparison of different sensors.

Sensor Mode	Resolution	Range	Durability	Core Benefits	Main Limitations	Applicable Conditions
Piezoresistive Sensor	Medium	Medium to wide	Medium (prone to aging/drift)	Low cost; easy flexible integration.	Sensitive to temperature and humidity; poor repeatability.	Short-term deployment (Greenhouses).
Capacitive Sensor	High	Medium	Medium (aging of dielectric materials)	High sensitivity; suitable for small forces.	Very sensitive to moisture; parasitic capacitance.	Dry environments (e.g., greenhouses).
Piezoelectric Sensor	High (dynamic)	Wide dynamic range	Relatively high	Very fast dynamic response.	Cannot measure static forces.	Grasping impact detection.
Fiber Bragg Grating (FBG) Sensor	Very high	Medium	Medium (adhesive joints are fragile)	High accuracy; immune to electromagnetic interference.	High cost; packaging difficulties.	Research-grade force control.
Hall Sensor	High	Medium	Very high (non-contact)	Pollution-resistant; high durability.	Relatively large volume.	Harsh environments (high dust and humidity).
Optical Sensor	High	Medium	Medium (optical path is easily contaminated)	High accuracy; fast response.	Sensitive to dust and water droplets.	Relatively clean environments.

**Table 7:** End-effectors field deployability checklist.

Engineering Item	Reason for Attention	Suggested Reporting Indicators	Suggested Design Implementation	Suggested Testing Method	Literature Evaluation Method
Dustproof and Waterproof	High humidity, muddy water, and dust can cause motors, sensors, and connectors to fail.	Standard greenhouse: IP54 minimum. Frequent washdowns: IP65 required.	Motor housing is sealed, fitted with waterproof connectors and cable glands.	After spraying/exposure to wet dust, conduct functional retesting to compare changes in success rate, response speed, and failure rate.	The report clearly specifies the level and includes testing: high; only describes the sealing design: medium; does not mention: low.
Easy to clean	Juice, soil, and pesticide residues can contaminate contact parts and affect food safety and reuse.	Report whether the contact piece is detachable and washable, whether it is resistant to routine disinfection, and whether cleaning dead zones can be avoided.	Suction cups, finger cots, etc. adopt quick-release structures; the contact surfaces feature rounded corners to minimize gaps and grooves.	Repeat contamination and check for residue after cleaning cycle.	With structure and cleaning validation: high; only qualitative description indicating easy cleaning: medium; not mentioned: low.
Easy to maintain	Field downtime directly affects continuous operation efficiency.	Report the replacement schedule for consumables such as suction cups, blades, and flexible finger cots.	Modular interface, design of quick-release structures such as buckles, magnetic attachments, and quick-release screws; standardization of consumables.	Record the replacement time for each individual and assess whether the connection accuracy decreases after repeated replacements.	With quantitative replacement time and repeated assembly verification: high; only stating that it is replaceable: medium; not mentioned: low.
Anti-aging	Ultraviolet radiation (UV), temperature differences, and pesticide corrosion can cause flexible materials to harden, crack, and experience a decline in performance.	Report on changes in hardness, stiffness, surface cracks, and success rate before and after accelerated aging.	Select UV-resistant and corrosion-resistant materials, separate design of flexible components and load-bearing framework.	Perform performance retesting after UV exposure, temperature and humidity cycling, and spraying/soaking in pesticide solution.	Accelerated aging test: high; discussion on material durability only: medium; no mention: low.
Aspiration prevention	In areas with dense foliage, it is easy to suck up leaves or experience local air leaks, leading to failure in grasping.	Reporting on the logic of mis-inhalation detection, determination threshold, and release strategy.	Vacuum pressure closed-loop control, short-term stability testing after adsorption, abnormal release detection, and secondary alignment.	Statistics on aspiration rate, release success rate, and recovery time after aspiration.	Detection and release closed-loop: high; only mentioned aspiration issue: medium; not mentioned: low.

Note: High, medium and low ratings indicate the level of detail reported in a given major study of the project, not the absolute performance ranking of end effectors.

## Data Availability

No new data were created or analyzed in this study.

## Appendix A

This paper adopted a systematic retrieval strategy. The retrieval databases are mainly from web of science, MDPI, Google Scholar, IEEE Xplore and ScienceDirect. The time range of the literature retrieval is 2010–2025. Around 2010, soft pneumatic grippers, vacuum cup end actuators and field test robots for agricultural picking design appeared. The previous research is mostly limited to rigid industrial fixtures in laboratory environments, which is not only of low research value, but also makes it difficult to deal with complex, unstructured environments. This stage was also selected because of the important development stage of agricultural end effectors. Search keywords mainly include: harvest or picking or fruit and vegetable harvest; end effector or clamp or soft clamp or suction or vacuum or cutting. The selection criteria were mainly English journal papers and conference papers, and empirical research on highly cited and open source data sets in the past five years; the research object is the end effector of agricultural picking robots, specifically describing and including the structure or operating mechanism of the end effector.

Criteria for exclusion were: unclear experimental design, insufficient sample size or lack of field validation data; the research content was non-picking task or non-end effector related, etc.; the structure of the end effector or the interaction mechanism with crops were not specifically described and eliminated. At the same time, only the study with the most complete information was reserved for repeated publications of the same study. Finally, 118 high-quality studies were included for systematic analysis.
